# Resilience and coping strategies of older adults in Hong Kong during COVID-19 pandemic: a mixed methods study

**DOI:** 10.1186/s12877-022-03009-3

**Published:** 2022-04-08

**Authors:** Siu-Ming Chan, Gary Ka-Ki Chung, Yat-Hang Chan, Roger Yat-Nork Chung, Hung Wong, Eng Kiong Yeoh, Jean Woo

**Affiliations:** 1 CUHK Institute of Health Equity, The Chinese University of Hong Kong, Hong Kong SAR, China; 2 Department of Social and Behavioural Sciences, City University of Hong Kong, Hong Kong SAR, China; 3 CUHK Institute of Ageing, The Chinese University of Hong Kong, Hong Kong SAR, China; 4 The Jockey Club School of Public Health and Primary Care, The Chinese University of Hong Kong, Hong Kong SAR, China; 5 Department of Social Work, The Chinese University of Hong Kong, Hong Kong SAR, China

**Keywords:** Worries about COVID-19, Social capital, Vulnerability, Subjective well-being, Community support

## Abstract

**Background:**

Despite the adverse physical health impact of COVID-19 on older adults, whether they are psychosocially vulnerable under the pandemic remains debatable. In this mixed methods study, we examined the psychosocial vulnerability of older adults relative to their younger counterparts and explored how they coped with the pandemic.

**Methods:**

From September to October 2020, 1067 adults in Hong Kong were randomly sampled and completed a telephone survey, whereas 10 older adults were recruited for individual interviews between September 2020 and April 2021. Quantitative measurements included subjective well-being, worries about COVID-19, and changes in social capital and social interaction since the pandemic. The transcribed qualitative data were closely read and summarized using thematic analyses.

**Results:**

Compared with younger adults, older adults tended to be less worried about COVID-19 infection and economic activity/livelihood, despite being slightly more worried about supplies of personal protective equipment. They also had better subjective well-being in terms of happiness and life satisfaction, with their social capital and social interaction less affected. In addition, five themes emerged from the qualitative interviews: (1) life philosophy; (2) economic security; (3) telecommunication; (4) role of community organizations and social workers; and (5) positive coping strategies.

**Conclusions:**

Older adults in this study showed better psychosocial well-being than their younger counterparts under the COVID-19 pandemic, which challenged the deeply rooted societal stereotype about the vulnerability of older adults. The stronger resilience for positive coping, technological assistance, and targeted government and community support may have protected older adults from distress during the pandemic.

## Background

The COVID-19 pandemic has been a global challenge since early 2020. Despite the differential size of local outbreaks ranging from thousands to millions across regions of the world, the sociodemographic patterns of the infected cases are comparable. One of the most consistent patterns is that older adults are commonly deemed as the high-risk group in terms of high incidence and mortality rates [1–4]. A review of data from European countries showed that more than 90% of the deaths due to COVID-19, as of May 2020, have been in adults aged 60 or above [5]. Since the introduction of COVID-19 vaccines, older adults may be facing extra worries and uncertainty about the protection and side effects of newly developed vaccines [6, 7]. Moreover, on top of the general social distancing policies, extra containment measures have been implemented in residential care homes [2], including restricted outdoor activities and visiting arrangements which exert a disproportionate impact on older adults. All the above issues have hence put older adults under the spotlight during the pandemic, with a general impression that they fared worse than their younger counterparts.

Undoubtedly, the existing scientific evidence shows that older adults are more physically vulnerable to COVID-19; however, they may not necessarily be at risk psychosocially under the pandemic. While the higher case fatality rate and containment measures may have imposed psychosocial distress on older adults, whether such potentially stressful events exert an adverse impact on their psychosocial health and well-being remains inconclusive. In particular, recent studies provided some clues that older adults may be no less resilient or less capable of coping under the pandemic compared with younger adults [8–12]. Some even suggested the potentially stronger resilience in older adults to cope with the COVID-19 stressors [7, 13, 14], with social support serving as a protective factor for psychosocial well-being for those with lower resilience [15]. Thus, the conventional impression of the psychosocial vulnerability of older adults under the pandemic may not be tenable especially in terms of psychosocial health and well-being.

In Hong Kong, older adults, particularly those living in residential care homes, are facing difficulties with social isolation during the pandemic. Despite the relatively low COVID-19 incidence rate, the containment measures are at least as stringent as in other world regions. Nonetheless, whether the potentially stressful events under the adversity of the pandemic would in turn lead to poorer psychosocial health and wellbeing could depend on a range of factors including one’s resilience, coping, and the level of social and community support. Hence, this mixed methods study aims to quantitatively and qualitatively examine whether older adults in Hong Kong are psychosocially more vulnerable compared with younger adults, and then explore how they cope with the COVID-19 pandemic.

## Methods

The present study adopted a mixed methods design to understand the psychosocial well-being and living experience of older adults in terms of resilience and coping strategies under COVID-19 pandemic. Both quantitative and qualitative research methods were used to collect related information via a population-based telephone survey and in-depth qualitative interviews by zoom or face to face interviews with older adults. Ethical approval was obtained from the Joint Chinese University of Hong Kong-New Territories East Cluster Clinical Research Ethics Committee in August 2020. All participants voluntarily joined the study with written informed consent. The interviews were recorded with the consent of participants, with the privacy and confidentiality ensured.

### Quantitative data collection

Quantitative data were collected from a random sample of households via a telephone survey. In order to minimize the sampling error, telephone numbers were first selected randomly from an updated telephone directory as seed numbers. The updated directory was composed of all the landline telephone numbers from Hong Kong residents in 2018, which was retrieved from an online website of a local telephone company. Another 3 sets of numbers were then generated using randomization of the last 2 digits in order to recruit the unlisted numbers. Duplicate numbers were then screened out, and the remaining numbers mixed in a random order to become the final sample. All participants were Hong Kong Chinese adults aged 18 or above. Upon successful contact with a target household, one eligible household member was selected using the last birthday method. The telephone survey was carried out by trained interviewers from September to October 2020. Among the 12,443 dialed telephone numbers, 10,555 were invalid of which 254 were non-residential lines, 4776 were fax lines/invalid lines, 1308 were cut off immediately, and 4217 were non-contacts after three attempts. Out of the 1888 answered calls, a final sample of 1067 adults was obtained with a response rate of 56.5%. To ensure the representativeness of our sample, proportional weighting of age group and sex was adopted to reduce the discrepancies between the surveyed adults and the general population with reference to the age and sex structure of the mid-2020 Hong Kong population obtained from the Census and Statistics Department of Hong Kong Government. The sociodemographic characteristics of the sample can be found in the Table 1.

**Table 1:** Weighted sociodemographic characteristics of the sample

	N	%
Gender		
Male	477	44.7
Female	590	55.3
Age		
18–39	353	33.1
40–59	389	36.5
≥ 60	325	30.4
Marital status		
Married/cohabit	718	67.4
Single/separated/divorces/widowed	348	32.6
Education		
Primary or below	182	17.2
Secondary	466	44.1
Post-secondary	94	8.9
Tertiary and above	314	29.7
Housing type		
Public rental housing	355	33.9
Public subsidized owned housing	156	14.9
Private rental housing	70	6.7
Privately owned housing	465	44.4

Several questions were asked in the questionnaire to explore the types and level of worries of the population. Regarding the impact of COVID-19, they were asked “What do you worry about specifically in terms of COVID-19?” With reference to the existing literature [16, 17], the items included being infected, economic activities and livelihood, supply of personal protective equipment (PPE), and the personal savings based on a five-point Likert scale from “Not worried at all (1)” to “very worried (5)”, where a higher score means a greater level of worry. The level of worries reflected the psychological vulnerability of individuals. In addition, four questions were asked to measure changes in social capital since the pandemic. Social capital refers to the social networks, social connection and trust among individuals [18, 19] Items included “cannot trust anyone even more”, “cannot ask for help from others even more”, “more unwilling to help others easily”, and “felt lonelier within a week”. Answers were recorded using a five-point Likert scale from “totally disagree (1)” to “totally agree (5)”, where a higher score means a lower social capital. Regarding changes in social interaction since the pandemic, respondents were asked “After the outbreak of COVID-19, how much did the meeting frequencies with the following members reduce?” Items included “family members and relatives who are not living together” and “friends” based on a five-point Likert scale from “reduced a lot (1)” to “increased a lot (5),” where a higher score means a better change in social interaction. Last, the subjective well-being of respondents was measured with reference to the guidelines of the Organisation for Economic Co-operation and Development (OECD) for measuring subjective well-being [20]. Respondents were asked to rate themselves on a scale from 0 to 10 for each of the following three questions: (1) “Overall, how satisfied are you with life as a whole these days?” (2) “Overall, to what extent do you feel the things you do in your life are worthwhile?” and (3) “How happy you felt yesterday?” The higher the respective scores, the better the subjective well-being our respondents have. The Cronbach’s alpha for worry, social capital, social interaction and subjective well-being are 0.686, 0.855, 0.793, 0.807, respectively, which showed moderate to high measurement reliability. The details of measurements had been denoted elsewhere [19, 21].

### Qualitative data collection

This study also adopted in-depth semi-structured interviews for qualitative investigation. Ten older adults were invited to share their living experience during COVID-19 pandemic. The strength of in-depth qualitative interview is that it provides more comprehensive understanding for human behavior and social phenomenon [22]. The result of qualitative study, though not generalizable, revealed the subjective views of individuals and the contextual background of participants which normally overlook in quantitative research [23]. The inclusion criteria were older adults aged 60 years or above who are able to speak in Cantonese and willing to share their living experience under COVID-19 pandemic. Purposeful sampling was used for participant recruitment with maximum variation in consideration of types of residence, education level, and family composition. This aimed at selecting information-rich cases to reach data saturation [24].

Data were collected through individual interviews by two trained researchers with a PhD degree between September 2020 and April 2021. Participants were referred by social workers in elderly homes and local non-governmental organizations (NGOs) who served older adults. A semi-structured interview guide was developed based on previous literature to study the impact of COVID-19 pandemic on older adults. It included open questions asking the participants about their daily experience, economic situation, social interaction, as well as physical and mental health conditions. Participants were also encouraged to share their feelings and thoughts about the COVID-19 pandemic. Each interview lasted for around 45 to 60 min and was conducted in Cantonese. All interviews were either audio-recorded for face-to-face interviews or video-recorded for Zoom interviews, which were then transcribed into text by trained student helpers.

### Statistical analysis

To examine the age-related differences in COVID-19 experience via the telephone survey, the mean scores of worries, social capital, social interaction, and subjective well-being were compared across age groups using ANOVA with their corresponding *p*-values and 95% confidence intervals. Apart from quantitative analyses, the thematic analysis approach, guided by Miles & Huberman [25], was also employed to explore the resilience and coping strategies of older adults via qualitative analyses. First, three researchers read the transcripts independently for several times to gain an overall understanding of their experience. Second, the researchers conducted open coding which summarized and extracted meaningful wordings. Codes were then further grouped into meaningful themes, clusters, and categories. Third, different opinions on the coding and themes were resolved by discussion within a group of experienced researchers. Credibility was established by data triangulation (i.e. checking data in field notes) and investigator triangulation (i.e. peer debriefing). Transferability was also achieved by considering the characteristics and experience of participants through in-depth interviews [26, 27].

## Results

### Quantitative analyses

The mean scores and standard deviations (SDs) of respondents’ worries, social capital, social interaction, and subjective well-being were summarized in Table 2. After weighting, among 1067 respondents, there were 353 cases (33.1%) aged 18 to 39, 389 cases (36.4%) aged 40 to 59, and 325 older adult cases (30.4%) aged 60 or above. Overall, older adults had lower level of worries about COVID-19 infection (mean = 2.95, SD = 1.16; *p*_ANOVA_ < 0.001) and economic livelihood (mean = 2.86, SD = 1.16; *p*_ANOVA_ < 0.001), but were slightly more worried about supplies of PPE (mean = 2.02, SD = 0.69; *p*_ANOVA_ = 0.002), compared with the other two younger groups. The difference of worries about personal savings among age group was not significant. In terms of social capital, generally the two younger groups had less social capital than older adults. Specially, the younger groups were less likely to trust anyone compared with older adults (mean = 1.81, SD = 0.80; *p*_ANOVA_ = 0.007). For social interaction, those aged 40 to 59 reduced interaction with families or relatives the most (mean = 1.60, SD = 0.60; *p*_ANOVA_ = 0.039) while older adults reduced interaction the least (mean = 1.71, SD = 0.59; *p*_ANOVA_ = 0.039). The difference of social interaction with friends among age groups was not significant. Regarding their subjective well-being, older adults consistently felt happier (mean = 7.21, SD = 1.45; *p*_ANOVA_ < 0.001), found the things they do in their life more worthwhile (mean = 7.20, SD = 1.48; *p*_ANOVA_ = 0.050), and were more satisfied with their life as a whole (mean = 7.25, SD = 1.49; *p*_ANOVA_ < 0.001) than their younger counterparts.

**Table 2:** Descriptive telephone survey results across age groups

	Aged 18–39 (***N*** = 353; 33.1%)	Aged 40–59 (***N*** = 389; 36.4%)	Aged ≥ 60 (***N*** = 325; 30.4%)	*p*_ANOVA_
	Means (SD)	Means (SD)	Means (SD)	
***Worry (1 = Not very worried; 5 = very worried)***				
Worry about being infected (Yourself / Family members)	3.34 (1.12)	3.42 (1.19)	2.95 (1.16)	<.001
Worry about economic activity/livelihood (e.g. Job lost / Salary reduction / Working time reduction)	3.17 (1.18)	3.27 (1.24)	2.86 (1.16)	<.001
Worry about supplies of personal protective equipment (e.g. face masks, hygiene products)	1.87 (0.52)	1.88 (0.72)	2.02 (0.69)	.002
Worry about personal savings	2.90 (1.10)	2.94 (1.18)	2.82 (1.08)	.368
***Social Capital (1 = totally disagree; 5 = totally agree)***				
I cannot trust anyone even more.	2.01 (0.91)	1.85 (0.86)	1.81 (0.80)	.007
I cannot ask for help from others even more.	2.03 (0.91)	1.90 (0.91)	1.88 (0.86)	.063
I am more unwilling to help others easily.	1.90 (0.86)	1.76 (0.80)	1.79 (0.82)	.055
I felt lonelier within a week.	2.16 (1.00)	2.10 (1.06)	2.10 (0.98)	.619
***Social interaction (1 = reduced a lot; 5 = increased a lot)***				
Meeting with family members / Relatives who are not living with you.	1.66 (0.56)	1.60 (0.60)	1.71 (0.59)	.039
Meeting with friends	1.46 (0.54)	1.41 (0.54)	1.49 (0.54)	.125
***Subjective Well-being (0 = not at all; 10 = experienced all of the time)***				
How happy you felt yesterday?	6.27 (1.50)	6.50 (1.58)	7.21 (1.45)	<.001
To what extent do you feel the things you do in your life are worthwhile?	6.92 (1.56)	7.12 (1.62)	7.20 (1.48)	.050
How satisfied are you with life as a whole these days?	5.93 (1.53)	6.32 (1.64)	7.25 (1.49)	<.001

### Qualitative analyses

Given the above quantitative results, we conducted qualitative interviews to further explore the possible reasons and mechanisms behind the resilience and coping strategies of older adults. The 10 participating older adults were aged from 60 to 91, five of whom were female (Table 3). Their education level ranged from primary or below to high school. Three of them were residents in elderly home whereas six of them lived in public rental housing, in addition to one living in a private cubicle. They had different types of income source, including Old Age Allowance, Comprehensive Social Security Assistance (CSSA), and support from children. During the individual interviews, five themes emerged as follows:

**Table 3:** Profile of participants in qualitative interviews

Participant	Sex/Age	Education	Marital Status	Housing type	No. of family members	Income Source
A	M/65	Primary or below	Married	Elderly Home	1	Comprehensive Social Security Assistance Scheme / Income from children
B	M/68	High School	Single	Elderly Home	1	Government Pension
C	F/91	Primary or below	Widowed	Elderly Home	1	Comprehensive Social Security Assistance Scheme
D	F/65	Junior High	Single	Public Rental Housing	1	Savings
E	F/77	Primary or below	Widowed	Public Rental Housing	1	Income from children / Other income sources
F	M/70	High School	Married	Public Rental Housing	4	Old Age Allowance / Income from children
G	M/72	High School	Widowed	Public Rental Housing	1	Comprehensive Social Security Assistance Scheme
H	F/80	Junior School	Married	Public Rental Housing	2	Old Age Allowance
I	F/66	Junior School	Widowed	Public Rental Housing	1	Old Age Allowance
J	M/60	Upper secondary	Separated	Cubicle	1	Savings

### Theme 1: life philosophy

It was believed that people were commonly worried about being infected by and dying of COVID-19. However, as reaching towards the end of life, the older adults we interviewed seemed to become less worried about death. Rather, many of them showed a sense of altruism during the interviews. They did not only show sincere care to their relatives and friends, but also to the general public and, in particular, the younger generation. Case C, an old lady living in elderly home, said she was not afraid of death because she was already very old. Another older adult, Case F, also expressed that he was not afraid of death.

*“(Are you worried under the pandemic?) I am worried about the younger generation. I am not afraid of death. I am old already. I am worried about my grandson getting sick and died. Die? I am not afraid. I will just close my eyes when I am going to die.”* (Case C, female, 91, elderly home)

*“(You said you keep on going restaurant. Someone says it is not suggested to go outside under the pandemic, what do you think?) My friends and I were okay. No problem about going outside. You know what older adults think….we are not that worried about death. We have already spent most of our life. What should we worry about? It would be fine if we wear a mask. No problem.”* (Case F, male, 70, public housing)

### Theme 2: economic security

While the working families, in general, are critically affected by the economic downturn due to job loss or underemployment under the COVID-19 pandemic, many older adults are retired and receiving social welfare from the government. Compared with those younger working adults, the financial situation of older adults appeared to be more stable. For example, Case F, a 70-year-old man, thought that the social welfare provided by the government made him feel secured under the pandemic.

*“(It seems that you are quite optimistic. Do you think so? And why?) Well, indeed I am not a typical optimistic person. One thing is crucial to me. It is economy security. There is no need to be rich, but economically stable. I usually tell my friends you can go gambling, but do not lose all your money. I mean, at least we should keep some money in hand. We are all older adults, we can get old age allowance from the government. This makes us feel safe.”* (Case F, male, 70, public housing)

Another retired older adult, Case B, also thought that he did not need to worry under the pandemic because he received stable income from the government.

*“The government gives me money to buy facial masks. (Social security?) I am a retired civil servant and I get pension from the government every month. That is why I do not have financial worry. (Do you think the economic downturn may affect your pension?) I don’t think so.”* (Case B, male, 68, elderly home)

### Theme 3: telecommunication

Although most governments implemented social distancing and isolation measures during the COVID-19 pandemic to protect people from infection, such measures may cause significant negative impacts on the social interaction and support among individuals. It was believed that older adults were at higher risk of social isolation as they may be less capable of using telecommunication to interact with others. Nevertheless, the ability of using telecommunication may depend on the educational level of older adults and also their social network. Some older adults were eager to learn new technologies. For example, Case I learnt how to use telecommunication via the elderly center and became more active than before. The impact of COVID-19 on social interaction of older adults may be compensated by using telecommunication.

*“(Do you feel lonely while staying at home?) Sometimes. (Did you participate in activities of the center less frequently?) Indeed, I joined the activities more frequently because I learnt how to use Zoom. (Oh, why and how did you learn?) Previously, I thought there were too many older adults in the center and I do not feel comfortable to stay there. But it is much better for me to join via Zoom. I can learn many things while staying at home. (Do you think this type of technology help you under the pandemic?) Sure, because of the pandemic, I learnt how to use YouTube and how to search information online. I can learn many things online. It really enriched my life.”* (Case I, female, 66, public housing)

### Theme 4: role of community organizations and social workers

During the COVID-19 pandemic, many community service centers and elderly centers were closed temporarily while some remained limited service. Many older adults mentioned the importance of elderly centers and social workers for maintaining their mental well-being during the pandemic.

*“(Do you think the elderly center is important to you?) If there were no elderly centers in the community, we older adults would be very hopeless and disappointed. We are getting old and there is no one to rely on. We can chat with each other in the centers and we have sustenance here. We want to spend our rest of life leisurely. The center and social workers are so important to us.”* (Case G, male, 72, public housing)

Some poor working older adults got unemployed and underemployed during the pandemic. They found it difficult to find a new job, especially as they are old. The NGOs and charity organizations played a role in providing timely material and financial support to the needy in addition to the government social welfare schemes.

*“Recently I do only earn a little, almost no savings… (So how did you use you money for daily living?) I even need to squeeze my expense on food…and I try to ask my friends about the community resource. You know, there are some NGOs, charity groups and social workers, and they will deliver some food coupons to the needy. Luckily I found them to help.”* (Case J, male, 60, cubicle)

### Theme 5: positive coping strategies

Sports and entertainment were found as common coping strategies for older adults to stay resilient against the negative impact induced by the pandemic. Some older adults exercised daily in order to keep physically and mentally healthy. ‘Tai-chi’ and ‘Qi-qong’ were common sports that older adults found them beneficial to their health. This also helped them become less worried about the COVID-19 pandemic.

*“(Do you play ‘Qi-qong’?) Yes, my master is famous in teaching ‘Qi-gong’, she taught us ‘lion roar’, do you hear this before? Really work! (Do you feel good?) Sure. After playing ‘lion roar’, I did not get sick for more than two to three years. It really helped me stay healthy and I keep playing this under the pandemic as well.”* (Case F, male, 70, public housing)

Playing music was another common strategy that older adults used to cope with their stress. For example, Case F play a Chinese musical instrument ‘Erhu’ every day and he felt much relaxed after playing music.

*“(Is your mood affected by the pandemic? What will you do?) Surely affected. When I have a bad mood, I will play Erhu. I am not good at playing musical instrument, but I really enjoy it. I also like singing, of course not very good. But this does help me relax.”* (Case F, male, 70, public housing)

## Discussion

This mixed methods study supported the counter-narrative to the deeply rooted societal stereotype on the vulnerability of older adults by providing both quantitative and qualitative evidence on the better-than-expected psychosocial well-being, resilience, and coping in older adults under the COVID-19 pandemic in Hong Kong. One assumption is that older adults were defenseless and assailable to the pandemic. However, this study highlights resilience within this group with regards personal coping strategies, socio-economic resources, and community and policy support.. Our quantitative results showed that older adults tended to be less worried about the impact of COVID-19 with better subjective well-being, and had more social capital and social interaction less reduced compared with their younger counterparts. Such findings could be supplemented and explained by our qualitative findings, which revealed that the resilience of older adults may have been enhanced by their life philosophy, economic security, improved digital literacy for telecommunication, social and community support, and positive coping strategies.

The observation that older adults fared no worse or even better than younger adults in terms of resilience and psychosocial coping under the pandemic has been portrayed in recent COVID-19 research. While we might expect a substantial decline in subjective well-being due to COVID-19 in older adults who are often deemed socially vulnerable, Kivi et al. [7] showed the opposite by reporting a stable level of life satisfaction and loneliness and improved self-rated health and financial satisfaction in Swedish older adults during the early phase of pandemic when compared to 5 years before the pandemic. In addition, Klaiber et al. [13] revealed that older adults were less concerned about the threats of COVID-19 especially in terms of their emotional well-being and financial situation. These findings appear to be in line with the strength and vulnerability integration model which postulated a better emotional well-being and emotional regulation with increased age [28].

Although the risk of infection and death is generally considered as one of the major worries under the pandemic, our results revealed that older adults were not as worried about contracting or dying of COVID-19 as most expected. The strong resilience of older adults in Hong Kong could at least be partially attributable to their life experience and age-related changes in life philosophy (see Theme 1). In addition to the possibility that older adults may simply become less afraid of death as they have already lived most of their life, the great deal of experience, expertise, and challenges throughout their lifetime has also been suggested to provide older adults with psychosocial strengths to go through the difficult times of the pandemic [14]. As one of the proposed psychosocial strengths, generativity for older adults enables them to be more aware of death of oneself and their loved ones, and thus become more prepared to envision the future of the next generations without them [14]. This explanation echoes with earlier research on the potential “death-denying” properties of generativity, which could in turn act as a buffer against death anxiety as people age [29]. Moreover, the accumulated life experience could lead to better coping capability in older adults [30, 31]. Specifically, Fuller and Huseth-Zosel [32] reported a high mean perceived coping level in older adults with the vast majority rating their coping positively, whereas Pearman et al. [33] suggested a more proactive coping could protect older adults from COVID-19 stress (see Theme 5). Positive coping strategies like exercising and spending leisure time on hobbies and entertainment could also bring joy and comfort and hence enhance psychosocial well-being in older adults [34, 35].

While older adults tended to be more resilient and skillful in coping, we should be aware of that they are indeed a heterogeneous group with varying vulnerability and responses to COVID-19 stress. Solely relying on individual resilience would inevitably leave the more vulnerable older adults out. Therefore, government and community support are indispensable to act as a buffer against the adverse impact of low individual resilience on psychosocial well-being [15] (see Theme 4). In Hong Kong, the social welfare schemes, including old age allowance, old age living allowance, pension benefits for retired civil servants, and more generally the comprehensive social security assistance scheme [36, 37], ensured a basic level of economic security in older adults and provided a safety net to those with low income (see Theme 2). Access to food, face masks and other personal hygiene equipment, although being of concern during the early phase of the pandemic, has also been facilitated by local NGOs and the strong community mobilization in Hong Kong [38, 39]. Apart from financial and material assistance, another major support by the government and NGOs during the pandemic was on the use of telecommunication. While older adults, especially those less educated, generally have lower digital literacy [40] and hence greater difficulty in maintaining online social networking, the community organizations for older adults took swift actions to offer training on telecommunication and online software to their service users since the pandemic (see Theme 3). At the later phase of the pandemic, the Social Welfare Department also deployed the government-initiated anti-epidemic fund and sponsored community organizations to open around 1000 temporary positions for young people to provide technological assistance to older adults, rehabilitation service users, and employees of relevant organizations [41]. In addition to the COVID-19 responses, the relatively mild COVID-19 impact on the social interaction in older adults in Hong Kong could have also been attributed to the continuing effort on digital inclusion in older adults by the government prior to the pandemic, including regular outreach programmes, advanced training, and the launch of a web-based learning portal on information and communications technologies [42]. Furthermore, beyond the government and community NGOs, the philanthropic sector played a pivotal role in filling the unmet needs and service gaps during the pandemic. For example, the Hong Kong Jockey Club mobilized over $987 million to support a wide variety of COVID-19 community programmes ranging from provision of anti-epidemic care packs and food assistance, support for e-learning at home, to projects on sustainable recovery from the prolonged impact of COVID-19 [43]. In particular, more than a quarter of the donation was specifically spent on the large-scaled support project for older adults under COVID-19 and programmes on facilities enhancement for pandemic preparedness at residential care homes. Altogether, the concerted effect across the government, community, and philanthropic sectors appears to enhance socio-economic resource of older adults and to have safeguarded their psychosocial health and well-being under the pandemic. The resilience of older adults accounted by coping strategies and community support was one critical element promoting the subjective well-being of them (see Theme 5).

Nevertheless, many older adults with functional needs depended on the day care services in the community. The day care centres, however, only provided limited services, if not closed, during the pandemic as the government did not classify community care as essential services as for the general out-patient clinics and hospital services. This resulted in serious deterioration of physical and cognitive functions in older adults, and in turn increased the stress and coping burden of their caregivers. In the future, the government should consider continuing such services operation with full personal protection equipment as for the residential care homes for the older adults. In addition, the no visiting policy for hospitals and residential care homes may also lead to distress and poorer quality of care in older adults. The personal care provided by family caregivers should be recognized as part of the services for dependent older patients, in particular those with dementia and varying extent of cognitive impairment.

There are several limitations in this study. First, for the quantitative part, the level of worries on COVID-19 may vary across phases of the pandemic. As the data was collected after the earliest phase of local outbreak, given the fluctuating nature of the pandemic, the level of worry of older adults may vary across different stages during the pandemic. Vaccination was not common during the period of data collection, and therefore was unlikely to impact on the results. Second, the quantitative study may be generalizable only to regions with relatively well COVID-19 control or with low incidence rate. Further research is needed to explore the resilience in older adults in regions being hardest hit. Third, for the qualitative part, given the small sample size and non-random sampling of interviewees, there may be heterogeneity in the experience, resilience, and coping strategies in older adults with different demographic, family, and cultural backgrounds. Therefore, the qualitative results may not be generalized. Fourth, the participants in the qualitative study mainly come from low socio-economic background and the views of older adults from middle or high socio-economic background may not be reflected. Finally, while the adverse psychosocial impact of the no visiting policy on hospital patients and residents of residential care homes could to some extent be alleviated by telecommunication technologies, some older adults suffered and died alone with few support from their relatives during their end-of-life period. These scenarios would not be captured by the design of this study.

To conclude, this study of the psychosocial well-being, worries, and social interaction of older adults, and their living experience during the COVID-19 pandemic showed that they tended to be more resilient than younger adults, and how their life philosophy, living styles, information technology, social welfare, and community services helped them cope with the pandemic. This study challenged the long-standing stereotype on the vulnerability of older adults, and provided possible themes on resilience and coping strategies of older adults against the pandemic for further research.

## Data Availability

The datasets generated and/or analysed during the current study are not publicly available due to consideration of confidentiality but are available from the corresponding author on reasonable request.

## References

[1] Chan EY, Lo ES, Huang Z, Kim JH, Hung H, Hung KK (2020). Characteristics and well-being of urban informal home care providers during COVID-19 pandemic: a population-based study. BMJ Open.

[2] Monahan C, Macdonald J, Lytle A, Apriceno M, Levy SR (2020). COVID-19 and ageism: how positive and negative responses impact older adults and society. Am Psychol.

[3] Strang P, Furst P, Schultz T (2020). Excess deaths from COVID-19 correlate with age and socio-economic status. A database study in the Stockholm region. Ups J Med Sci.

[4] Wu Z, McGoogan JM (2020). Characteristics of and important lessons from the coronavirus disease 2019 (COVID-19) outbreak in China: summary of a report of 72314 cases from the Chinese Center for Disease Control and Prevention. JAMA..

[5] Calderon-Larranaga A, Dekhtyar S, Vetrano DL, Bellander T, Fratiglioni L (2020). COVID-19: risk accumulation among biologically and socially vulnerable older populations. Ageing Res Rev.

[6] Soiza RL, Scicluna C, Thomson EC (2021). Efficacy and safety of COVID-19 vaccines in older people. Age Ageing.

[7] Kivi M, Hansson I, Bjalkebring P (2021). Up and about: older Adults' well-being during the COVID-19 pandemic in a Swedish longitudinal study. J Gerontol B Psychol Sci Soc Sci.

[8] Riehm KE, Brenneke SG, Adams LB, Gilan D, Lieb K, Kunzler AM (2021). Association between psychological resilience and changes in mental distress during the COVID-19 pandemic. J Affect Disord.

[9] Kowal M, Coll-Martin T, Ikizer G, Rasmussen J, Eichel K, Studzinska A (2020). Who is the Most stressed during the COVID-19 pandemic? Data from 26 countries and areas. Appl Psychol Health Well Being.

[10] Kimhi S, Marciano H, Eshel Y, Adini B (2020). Resilience and demographic characteristics predicting distress during the COVID-19 crisis. Soc Sci Med.

[11] Glowacz F, Schmits E (2020). Psychological distress during the COVID-19 lockdown: the young adults most at risk. Psychiatry Res.

[12] Young NA, Waugh CE, Minton AR, Charles ST, Haase CM, Mikels JA (2021). Reactive, Agentic, apathetic, or challenged? Aging, emotion, and coping during the COVID-19 pandemic. Gerontologist..

[13] Klaiber P, Wen JH, DeLongis A, Sin NL (2021). The ups and downs of daily life during COVID-19: age differences in affect, stress, and positive events. J Gerontol B Psychol Sci Soc Sci.

[14] Lind M, Bluck S, McAdams DP (2021). More vulnerable? The life story approach highlights older People's potential for strength during the pandemic. J Gerontol B Psychol Sci Soc Sci.

[15] Kim JH, Sumerlin TS, Goggins WB, Kwong EMS, Leung J, Yu B (2021). Does low subjective social status predict cognitive decline in Chinese older adults? A 4-year longitudinal study from Hong Kong. Am J Geriatr Psychiatr.

[16] Barber SJ, Kim H (2021). COVID-19 worries and behavior changes in older and younger men and women. J Gerontol B Psychol Sci Soc Sci.

[17] Hidaka Y, Sasaki N, Imamura K, Tsuno K, Kuroda R, Kawakami N (2021). Changes in fears and worries related to COVID-19 during the pandemic among current employees in Japan: a 5-month longitudinal study. Public Health.

[18] Woolcock M (2001). The place of social Capital in Understanding Social and Economic Outcome. Can J Policy Res.

[19] Chan SM, Chung GK, Chan YH, Woo J, Yeoh EK, Chung RY, et al. The mediating role of individual-level social capital among worries, mental health and subjective well-being among adults in Hong Kong during the COVID-19 pandemic. Curr Psychol. 2021:1–11. 10.1007/s12144-021-02316-z. 10.1007/s12144-021-02316-z PMC8459135 34580570

[20] OECD (2013). OECD Guidelines on Measuring Subjective Well-being.

[21] Chan SM, Wong H. Housing and Subjective Well-Being in Hong Kong: a structural equation model. Appl Res Qual Life. Forthcoming 2022. https://link.springer.com/article/10.1007/s11482-021-10000-4.

[22] Hesse-Biber SN (2010). Mixed methods research : merging theory with practice.

[23] Flick U (2000). An introduction to qualitative research. Eur J Inf Syst.

[24] Francis JJ, Johnston M, Robertson C, Glidewell L, Entwistle V, Eccles MP (2010). What is an adequate sample size? Operationalising data saturation for theory-based interview studies. Psychol Health.

[25] Miles MB, Huberman AM (1994). Qualitative data analysis : an expanded sourcebook.

[26] Korstjens I, Moser A (2018). Series: practical guidance to qualitative research. Part 4: trustworthiness and publishing. Eur J Gen Pract.

[27] Shenton AK (2004). Strategies for ensuring trustworthiness in qualitative research projects. Educ Inf.

[28] Charles ST (2010). Strength and vulnerability integration: a model of emotional well-being across adulthood. Psychol Bull.

[29] Major RJ, Whelton WJ, Schimel J, Sharpe D (2016). Older adults and the fear of death: the protective function of Generativity. Can J Aging.

[30] Aldwin CM, Igarashi H, Bengston V, Settersten RA (2016). Coping, optimal aging, and resilience in a sociocultural context. Handbook of theories of aging.

[31] Neupert SD, Neubauer AB, Scott SB, Hyun J, Sliwinski MJ (2019). Back to the future: examining age differences in processes before stressor exposure. J Gerontol B Psychol Sci Soc Sci.

[32] Fuller HR, Huseth-Zosel A (2021). Lessons in resilience: initial coping among older adults during the COVID-19 pandemic. Gerontologist..

[33] Pearman A, Hughes ML, Smith EL, Neupert SD (2021). Age differences in risk and resilience factors in COVID-19-related stress. J Gerontol B Psychol Sci Soc Sci.

[34] Carriedo A, Cecchini JA, Fernandez-Rio J, Mendez-Gimenez A (2020). COVID-19, psychological well-being and physical activity levels in older adults during the Nationwide lockdown in Spain. Am J Geriatr Psychiatry.

[35] Whitehead BR, Torossian E (2021). Older Adults' experience of the COVID-19 pandemic: a mixed-methods analysis of stresses and joys. Gerontologist..

[36] Social Welfare Department (2021). Comprehensive Social Security Assistance (CSSA) Scheme.

[37] Social Welfare Department (2021). Social Security Allowance (SSA) Scheme.

[38] Wan KM, Ho LK, Wong NW, Chiu A (2020). Fighting COVID-19 in Hong Kong: the effects of community and social mobilization. World Dev.

[39] Yuen S, Cheng EW, Or NHK, Grepin KA, Fu KW, Yung KC, et al. A tale of two city-states: a comparison of the state-led vs civil society-led responses to COVID-19 in Singapore and Hong Kong. Glob. Public Health. 2021;16(8-9):1283-303. 10.1080/17441692.2021.1877769 33592151

[40] Latulippe K, Hamel C, Giroux D (2017). Social health inequalities and eHealth: a literature review with qualitative synthesis of theoretical and empirical studies. J Med Internet Res.

[41] Labour and Welfare Bureau (2020). Gerontech and Innovation Expo.

[42] Legislative Council (2020). The Chief Executive’s 2020 Policy Address, Policy Initiatives of Innovation and Technology Bureau.

[43] The Hong Kong Jockey Club Charities Trust (2020). COVID-19 Community Support Initiatives.

